# Mitochondrial Antioxidants and the Maintenance of Cellular Hydrogen Peroxide Levels

**DOI:** 10.1155/2018/7857251

**Published:** 2018-07-02

**Authors:** Ryan J. Mailloux

**Affiliations:** Department of Biochemistry, Memorial University of Newfoundland, St. John's, NL, Canada

## Abstract

For over 40 years, mitochondrial reactive oxygen species (ROS) production and balance has been studied in the context of oxidative distress and tissue damage. However, research over the past decade has demonstrated that the mitochondria have a more complicated relationship with ROS. Superoxide (O_2_^•−^) and hydrogen peroxide (H_2_O_2_) are the proximal ROS formed by the mitochondria, and the latter molecule is used as a secondary messenger to coordinate oxidative metabolism with changes in cell physiology. Like any other secondary messenger, H_2_O_2_ levels need to be regulated through its production and degradation and the mitochondria are enriched with the antioxidant defenses required to degrade ROS formed by nutrient oxidation and respiration. Recent work has also demonstrated that these antioxidant systems also carry the capacity to clear H_2_O_2_ formed outside of mitochondria. These observations led to the development of the postulate that the mitochondria serve as “ROS stabilizing devices” that buffer cellular H_2_O_2_ levels. Here, I provide an updated view on mitochondrial ROS homeostasis and discuss the “ROS stabilizing” function of the mitochondria in mammalian cells. This will be followed by a hypothetical discussion on the potential function of the mitochondria and proton motive force in degrading cellular H_2_O_2_ signals emanating from cytosolic enzymes.

## 1. Introduction

ROS genesis by the mammalian mitochondria relies on the same electron transfer pathways that are also involved in nutrient oxidation and the biosynthesis of ATP. Electrons mobilized from the combustion of carbon are transferred to complexes I and II of the respiratory chain through the carriers NADH and succinate, respectively. After entry into the electron transport chain (ETC), electrons are ferried through the ubiquinone (UQ) pool and complex III to complex IV, reducing molecular oxygen (O_2_) to water [1]. Electron transfer through this chain is a thermodynamically favorable process and coupled with the pumping of protons by complexes I, III, and IV [2]. This creates an electrochemical difference of protons across the mitochondrial inner membrane (MIM), called a proton motive force (PMF), that is used by complex V to make ATP [2]. After its production, ATP is exported into the cytosol through the ADP/ATP translocase, also known as adenine nucleotide translocator (ANT), to perform useful “work” in the cell [3]. The proton gradient formed by the flux of electrons through the respiratory chain is also used for the selective uptake of solutes and proteins into the matrix. Proton return to the matrix also plays a critical role in the regulation of ROS levels. For instance, proton return through nicotinamide nucleotide transhydrogenase (NNT) is required for the provision of NADPH, a vital component of H_2_O_2_-degrading antioxidant systems [4].

**Figure 1:**
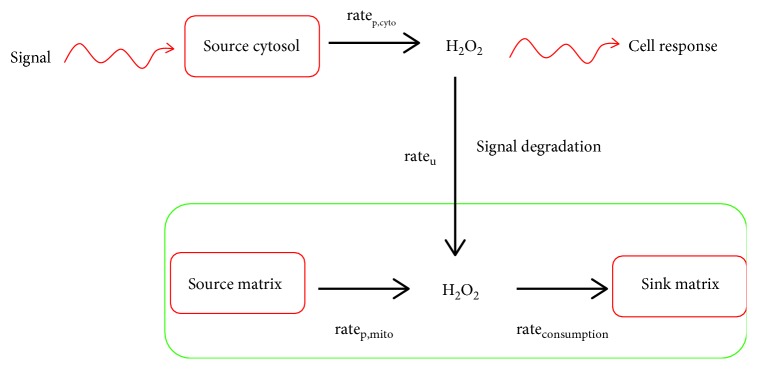
Mitochondrial are a sink for cellular hydrogen peroxide. The function of mitochondria as a cellular ROS stabilizer depends on the rate of H_2_O_2_ production (rate_p,mito_) and consumption (rate_consumption_). Accumulation of cellular H_2_O_2_ serves as an important signal, which can be desensitized by mitochondrial antioxidant defenses. The rate of H_2_O_2_ uptake by the mitochondria (rate_u_) is dependent on the redox buffering capacity of the matrix.

Mitochondria are equipped with antioxidant defenses to quench ROS [5]. However, these defenses are not used exclusively to clear ROS formed by nutrient metabolism and respiration. Several studies have demonstrated that matrix antioxidant defenses can also quench extramitochondrial H_2_O_2_ [6–8]. Clearance of extramitochondrial H_2_O_2_ depends on the redox buffering capacity of the matrix which is influenced by the availability of ROS and NADPH. Therefore, the degradation of cellular H_2_O_2_ by the mitochondria (rate of uptake, rate_u_) depends on (1) rate of mitochondrial H_2_O_2_ production (rate_p,mito_) and (2) the rate of mitochondrial H_2_O_2_ degradation (rate_consumption_) (Figure 1). Here, I review our current understanding of how the mitochondria buffer cellular H_2_O_2_ using the glutathione (GSH), thioredoxin (TRX), and catalase systems. I also discuss how this buffering capacity relies on mitochondrial respiration for the provision of NADPH through NNT. Finally, I elaborate on how this H_2_O_2_ buffering function of the mitochondria can potentially quench redox signals emanating from cytosolic ROS producers in response to physiological stimuli (Figure 1).

## 2. Mitochondrial H_2_O_2_ Homeostasis

### 2.1. Sources of Mitochondrial ROS

Mitochondria can contain up to 12 potential sources of ROS associated with nutrient oxidation (Table 1). These individual sites of production can be classified into two groups; the NADH/NAD^+^ isopotential group and the UQH_2_/UQ isopotential group (Table 1). The ROS-producing properties of these different enzymes have recently been reviewed extensively [9, 10]. ROS producers that fall in the former group generate O_2_^•−^/H_2_O_2_ in the presence of NADH. Group two enzymes on the other hand use the UQH_2_ pool to make ROS (Table 1) [10]. Some of these enzymes are high capacity sites for ROS release whereas others have low rates of production. In addition, which enzyme serves as a high capacity site for production can vary between different tissues, which may be related to substrate availability and mitochondrial bioenergetics (Table 1). For example, *α*-ketoglutarate dehydrogenase complex (KGDH), pyruvate dehydrogenase complex (PDH), and complex III account for ~90% of the ROS released by liver mitochondria (Table 1) [8]. Complexes I and III and to a lesser extent complex II display high rates for ROS production in heart mitochondria (Table 1) [11]. It has also been documented that KGDH and PDH produce ROS in cardiac mitochondria, with the former generating more than the latter [12]. However, it has been recently estimated that KGDH and PDH make minor contributions to overall ROS release from cardiac mitochondria [11]. Finally, complexes I, II and III, KGDH and PDH, and *sn*-glycerol-3-phosphate dehydrogenase (G3PDH) serve as high capacity sites in the muscle and KGDH and PDH produce ~8x and ~4x more ROS than complex I in these mitochondria [10]. The rate of production from these sites can vary considerably in response to different physiological conditions, which can affect the strength and duration of the H_2_O_2_ signal. The concentration and type of the substrate being oxidized and concentration and redox state of the electron-donating site are the most important factors that influence the rate of ROS release from the mitochondria [10]. Other factors like membrane potential strength, NADH availability, allosteric regulators, or posttranslational modifications, like protein S-glutathionylation, also affect ROS production [13, 14]. Conditions that mimic rest or exercise in the muscle have been shown to dictate which sites form the most ROS in muscle mitochondria, which can affect how much H_2_O_2_ is released into the extramitochondrial space [15]. This is associated with variations in the availability of different substrates and ions that ultimately influence the rate of ROS release from these sites [15]. Overall, up to 12 sites of production can contribute to the overall rate for ROS production (rate_p,mito_) in the mitochondria (Figure 1). In addition, rate_p,mito_ depends on the bioenergetics of the mitochondria and can vary in response to different physiological cues.

### 2.2. Antioxidant Defense Systems

The overall concentration of mitochondrial H_2_O_2_ also depends on the rate of its consumption (rate_consumption_) (Figure 1). The routine degradation of H_2_O_2_ is vital for controlling its secondary signaling properties and preventing oxidative distress. Mitochondrial ROS producers, like KGDH, PDH, and complexes I and II, have been shown to produce a mixture of O_2_^•−^ and H_2_O_2_, which is related to the free radical properties of flavin nucleotides [12, 16]. Recent estimates indicate that ~75% of the total ROS released by PDH and KGDH is H_2_O_2_ [17, 18]. However, mitochondria also form O_2_^•−^ nonetheless which needs to be removed rapidly to avoid the deactivation of Fe-S cluster-containing proteins. Mitochondria contain two superoxide dismutase isozymes (SOD), MnSOD (matrix-bound) and Cu/ZnSOD (intermembrane space) that catalyze the dismutation of two O_2_^•−^ to H_2_O_2_ [13]. SOD is highly concentrated in the mitochondria (10 *μ*M) and displays rapid kinetics (*k* ≈ 1.8 × 10^9^ M^−1^ s^−1^), maintaining the concentration of O_2_^•−^ at ~10–200 pM [13]. Therefore, although mitochondria do produce O_2_^•−^ via the univalent reduction of O_2_, the dominant ROS in the matrix environment is H_2_O_2_.

Mitochondria are equipped with two main H_2_O_2_ degrading pathways, the GSH and TRX2 systems [19]. Liver and cardiac mitochondria have also been found to contain catalase, which plays an important role in eliminating H_2_O_2_ [8, 20]. In contrast to catalase, the GSH and TRX2 systems require the reductive power of NADPH to eliminate ROS. The glutathione system relies on the oxidation of two GSH molecules in the presence of H_2_O_2_-forming glutathione disulfide (GSSG), a reaction catalyzed in the mitochondria by glutathione peroxidase (GPX). Mitochondria contain two GPX isozymes, GPX1 and GPX4. Both isozymes catalyze the sequestration of H_2_O_2_ with high efficiency (*k*_GPX1_ ≈ 6 × 10^7^ M^−1^ s^−1^ and *k*_GPX4_ ≈ 3 × 10^6^ M^−1^ s^−1^) [21]. Restoration of GSH levels after a round of H_2_O_2_ clearance is catalyzed by glutathione reductase (GR) in the presence of NADPH. The TRX system utilizes the catalytic activity of peroxiredoxin (PRX), which contains a peroxidatic cysteine (Cys_P_) in its active site to sequester H_2_O_2_ [22]. The oxidized Cys_P_ is resolved by a neighboring cysteine residue (resolving cysteine, Cys_R_) forming an intermolecular disulfide bridge. In the matrix, PRX3 and PRX5 are responsible for catalyzing these reactions (*k*_PRX3_ ≈ 2 × 10^7^ M^−1^ s^−1^ and *k*_PRX5_ ≈ 3 × 10^5^ M^−1^ s^−1^) [21]. In the matrix of the mitochondria, the reductive power stored in TRX2 reactivates PRX3 or 5 through a simple disulfide exchange reaction [23]. TRX2 is then reactivated by thioredoxin reductase-2 (TR2) in the presence of NADPH. Mitochondria have also been reported to use *α*-keto acids to spontaneously eliminate H_2_O_2_ [24]. Unfortunately, *α*-keto acids display slow kinetics for H_2_O_2_ degradation [25]. In addition, millimolar amounts are required to quench H_2_O_2_. Pyruvate has been reported to accumulate to ~0.5 mM but *α*-ketoglutarate typically occurs in the low *μ*M range [26]. By contrast, GPX1 and PRX3 occur at ~2 *μ*M and ~60 *μ*M, respectively, in the matrix and display high rates for H_2_O_2_ elimination. Therefore, it is unlikely that *α*-keto acids make any real contribution to H_2_O_2_ removal *in vivo*.

The contributions of the GSH and TRX2 systems to H_2_O_2_ elimination has been enthusiastically debated for several years. Competitive kinetic analyses contended that PRX accounts for ~90% of the H_2_O_2_ removal in the mitochondria [21]. Moreover, genetic ablation of the *Trx2* gene is embryonically lethal whereas loss of the *Gpx1* and *Gpx4* genes only sensitizes cells towards oxidative distress [27]. However, it needs to be emphasized that GPX1 and 4 are used exclusively for the elimination of H_2_O_2_ and lipid hydroperoxides whereas TRX2 is required to reduce disulfide bridges in a multitude of enzymes including ribonucleotide reductase. In addition, the impact of ablating the *Prx3* gene has not been examined and recent work utilizing cell lines has demonstrated that both systems are equally important at eliminating H_2_O_2_ [28]. Recent work has also found that the TRX2 system may fulfill a secondary function in eliminating H_2_O_2_ formed by respiration [8]. Indeed, Slade et al. found that only GSH and catalase were involved in eliminating H_2_O_2_ in mouse liver mitochondria whereas the TRX2 system made a negligible contribution [8]. Similar observations were made with rat liver mitochondria [29]. In fact, Rigobello et al. showed that the TRX2 system contributed to H_2_O_2_ elimination only when ROS was overproduced by liver mitochondria following treatment with the electron transport chain blocker, antimycin A [29]. These findings, however, do contradict studies conducted with muscle mitochondria. In two separate studies, it was demonstrated that the TRX2 system is integral for eliminating H_2_O_2_ formed by respiration [7, 30]. In addition, the GSH system was also found to play an important part in H_2_O_2_ removal in skeletal muscle mitochondria [7]. Similar observations were also made with neural mitochondria and skeletal muscle fibers [6, 30]. Although speculative, it is possible that different tissues rely on different systems for the elimination of H_2_O_2_. Hepatocytes are routinely exposed to high ROS due to their normal physiological functions and thus may rely on catalase and GSH to maintain the steady-state level of H_2_O_2_ with the TRX2 system serving as the third line of defense in case levels get too high. As indicated above, TRX2 is vital for reducing disulfide bridges formed in various proteins including PRX enzymes. Therefore, it is possible that liver cells may reserve TRX2 for the reduction of disulfide bonds and utilize it for antioxidant defense when other systems are overwhelmed by H_2_O_2_. By contrast, muscle and neural mitochondria are not routinely exposed to high ROS like hepatocytes and may simply utilize the GSH and TRX2 systems for H_2_O_2_ elimination.

### 2.3. Provision of NADPH and the Importance of Transhydrogenase

Antioxidant defenses depend on the reductive power stored in NADPH. Therefore, H_2_O_2_ degradation ultimately relies on the provision of NADPH. Mitochondria contain an entire suite of NADPH-producing enzymes that support antioxidant defenses. The complement of enzymes involved in producing NADPH in the matrix of mitochondria includes malic enzyme (ME), glutamate dehydrogenase (GDH), NADP^+^-dependent isocitrate dehydrogenase (IDH2), and NNT [31, 32]. It has also been found that mitochondria contain a matrix-associated glucose-6-phosphate dehydrogenase (G6PDH) isozyme, which has been speculated to play a role in the production of mitochondrial ribose sugars for nucleotide biosynthesis [33]. Unlike the other NADPH producers, which simply couple the oxidation of carbon to the reduction of NADP^+^, NNT actually assembles in the mitochondrial inner membrane and couples proton return to the matrix to the transfer of a hydride from NADH to NADP^+^ [34].

Work over the past decade has demonstrated that NNT is an important NADPH supplier in the mitochondria. This importance was first recognized when it was discovered that the C57Bl/6J mouse strain carries a loss-of-function variance in the *Nnt* gene [35]. These mice are glucose intolerant and have a glucocorticoid deficiency [36, 37]. Variances in the *Nnt* gene in humans are also associated with familial glucocorticoid deficiency [38]. The observation that the loss of the *Nnt* gene results in glucocorticoid deficiency is intriguing from a redox signaling perspective since it was recently found that mitochondrial ROS signals play a critical role in glucocorticoid biosynthesis and that oxidative distress hinders steroidogenesis [39]. Loss of NNT sensitizes mitochondria to oxidative distress, increases ROS release, and induces abnormalities in mitochondrial redox buffering capacity [32]. Deletion of the *Nnt* gene diminishes NADPH recovery time in liver mitochondria challenged with *tert*-butyl hydroperoxide [31]. In addition, there are no compensatory increases in the activities of IDH2, ME, or GDH in liver mitochondria from mice homozygous or heterozygous for the *Nnt* gene unless their cognate substrates are supplied at high concentrations [31, 32]. Loss of NNT also leads to spontaneous NADPH oxidation and sensitizes the mitochondria to permeability pore opening [32]. In addition, substrate supply plays an integral role in driving NADPH formation by NNT. It has been estimated that NNT accounts for most of the NADPH production in the mitochondria. This, however, depends on the bioenergetic state of the mitochondria [31]. For instance, up to 100% of the total NADPH can be produced by NNT in the mitochondria operating under state 4 respiratory conditions [31]. Induction of state 3 respiration diminishes the contribution of NNT towards NADPH production (accounting for ~50% or lower) [31]. At this point, it should be emphasized that state 3 respiratory conditions are highly artificial and do not represent a natural bioenergetic state of the mitochondria in cells. It has been estimated that the mitochondria actually operate between state 3 and 4 respiration in neural and muscle tissue and cultured cells (referred to as state_apparent_ to reflect the intermediary state of respiration in cells) [40–42]. Therefore, NNT is likely to be the major supplier for NADPH under physiological conditions. Finally, collapsing the mitochondrial membrane potential abolishes the NADPH-producing activity of NNT [31]. In fact, in one intriguing study, it was observed that collapsing the membrane potential reverses NNT [4]. In this case, it was found that it transfers a hydride from NADPH to NAD^+^ resulting in the pumping of protons into the intermembrane space [4]. This results in the depletion of mitochondrial NADPH, leaving cells vulnerable to oxidative attack. This also demonstrates the importance of NNT in maintaining the NADPH pool since its reversal results in oxidation of ROS clearing systems culminating with oxidative damage and development of heart disease.

Other sources of NADPH can, to a certain degree, compensate for the loss of NNT. C57Bl/6J mice are still viable regardless of the loss-of-function mutation for the *Nnt* gene. In addition, liver mitochondria from NNT knockout mice can maintain normal NADPH levels if isocitrate, malate, or glutamate are added to the reaction mixtures [31]. However, as noted above, compensation by these other NADPH-forming enzymes is limited [31]. Other pathways can also serve as NADPH suppliers in the mitochondria. Matrix-associated G6PDH was found to be an important source of NADPH in cultured myoblasts, human myotubes, and HEK293 cells cultured in a high glucose medium [33]. By contrast, exposure of cells to a low glucose medium rich in Krebs cycle-linked substrates upregulates IDH2 [33]. Therefore, mitochondria do have the ability to use various carbon sources to sustain NADPH production. However, it is likely that NNT is the major supplier *in vivo* since it utilizes a central and sustainable energy source, the proton gradient, to form NADPH.

## 3. Mitochondria as a Sink for Cellular Hydrogen Peroxide

**Figure 2:**
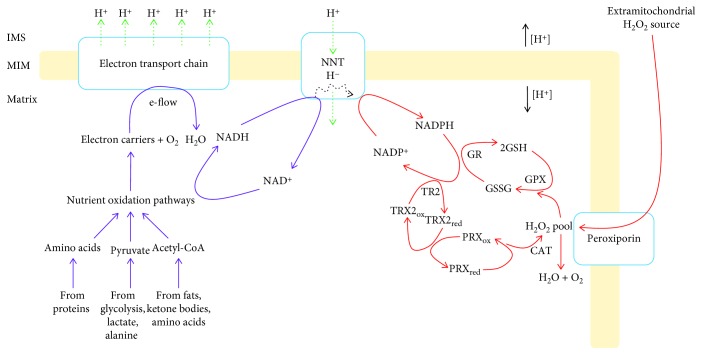
Nicotinamide nucleotide transhydrogenase (NNT) plays a central role in clearing cellular hydrogen peroxide. The combustion of carbon yielded from the metabolism of different nutrients generates electron carriers that are oxidized by respiratory chain enzymes. The electrons liberated by carrier oxidation results in the pumping of protons into the intermembrane space. Protons are returned to the matrix through NNT which powers the transfer of a hydride from NADH to NADP^+^, forming NADPH. Hydrogen peroxide generated in the cytoplasm is imported into the matrix by peroxiporin and then degraded by three different antioxidant pathways. NADPH is used to reduce oxidized GSH and TRX2 after a round of H_2_O_2_ elimination. Catalase can also remove H_2_O_2_.

Because the mitochondria are abundant in mammalian cells and enriched in antioxidant defenses, these organelles can serve as an ideal sink for cytosolic or extracellular H_2_O_2_. The rate for H_2_O_2_ uptake by the mitochondria depends on its redox buffering capacity (Figure 1). The capacity of mammalian mitochondria to clear H_2_O_2_ from the surrounding environment was initially proposed by Jezek and Hlavata in 2005 [43]. In this seminal article, it was hypothesized that the mitochondria can quench cytosolic H_2_O_2_ when the rate for ROS release from the respiratory chain (or potentially other sources) was low [43]. This would maintain mitochondrial redox buffering capacity in a more reduced state promoting clearance of H_2_O_2_ formed in the cytosol or extracellular milieu. The authors based this hypothesis on findings showing that uncoupling the PMF diminishes ROS release from the respiratory chain, allowing the clearance of H_2_O_2_ outside of the mitochondria [43]. These concepts were then tested with brain mitochondria where it was found that matrix antioxidant defenses can stabilize the steady-state concentration of H_2_O_2_ in the surrounding medium [6]. Through a series of well-designed experiments, it was demonstrated that the mitochondria serve as a “dampening device” for ROS, sequestering H_2_O_2_ when it is at a higher than normal concentration in the surrounding medium (Figure 2) [6]. Another key finding from this study was that the “ROS-stabilizing” feature of the mitochondria relied on substrate oxidation and respiration (Figure 2) [6]. This would allow for the provision of NADPH, a key ingredient for the maintenance of antioxidant defenses. The concept that the mitochondria can clear H_2_O_2_ from the surrounding medium was also demonstrated with muscle and liver mitochondria. Several studies found that rat muscle mitochondria can quench H_2_O_2_ from the surrounding buffer using both the GSH and TRX2 systems [7, 30]. It is also possible that catalase also aids in eliminating H_2_O_2_ external to muscle mitochondria as well [44]. The GSH and TRX2 systems play key roles in maintaining the steady-state level of extramitochondrial H_2_O_2_ levels in rat and mouse liver mitochondria [8]. Slade et al. also showed that catalase plays an integral role in eliminating extramitochondrial H_2_O_2_ when it is in excess [8]. Indeed, liver mitochondria isolated from C57Bl/6N mice were able to clear 2 *μ*M H_2_O_2_ within 2.5 minutes and catalase comprised ~55–60% of this quenching activity [8]. At lower levels, GSH and TRX2 systems were crucial at maintaining H_2_O_2_ steady-state levels (sub *μ*M range) outside of the mitochondria [8]. The caveat to the studies described above is that this H_2_O_2_ clearance feature was tested *in vitro* bringing into question the physiological relevance of mitochondria as cellular ROS-dampening devices. However, a recent study demonstrated that the mitochondria retain their capacity to clear cytosolic H_2_O_2_ in cardiac myoblasts [28]. In this seminal study, different mitochondrial antioxidant defenses and NADPH producers were knocked down to assess if the mitochondria are required to maintain the steady-state concentration of cytosolic H_2_O_2_. The authors found that maintenance of the cytosolic redox environment is highly dependent on mitochondrial H_2_O_2_ buffering capacity [28]. Moreover, it was documented that mitochondrial antioxidant defenses play a central role in clearing cellular H_2_O_2_. In addition, it was shown that this capacity is highly dependent on mitochondrial substrate oxidation and NADPH production by NNT [28]. Taken together, mitochondria have an innate ability to take up and degrade H_2_O_2_, a feature that depends on the provision of NADPH by NNT.

The redox code is defined as a “*set of principles that describes the spatiotemporal positioning, in terms of the redox state, of the nicotinamide pool (NAD^+^ and NADP^+^*) *and thiols/disulfides relative to the redox proteome in biological systems*” [45]. At its core, the redox code is influenced by the rate of H_2_O_2_ production and consumption, both of which rely on nutrient oxidation and the proton motive force [45]. One critical observation made regarding the H_2_O_2_-clearing abilities of the mitochondria is that it relies on substrate catabolism and respiration. The catabolism of carbon is required for the provision of NADPH and the maintenance of mitochondrial redox buffering networks in a reduced/active state. It is, therefore, also important to consider the source of this NADPH. Dey et al. demonstrated that knocking down IDH2, ME, or NNT does compromise the H_2_O_2_ quenching capacity of the mitochondria [28]. However, it was also observed that NNT was the largest contributor towards the degradation of H_2_O_2_ [28]. This agrees with the *in vitro* studies conducted with liver mitochondria that demonstrated that NNT is the chief supplier, and ME and IDH2 make minor contributions to the NADPH pool in the matrix [31]. Moreover, a study that was conducted with permeabilized muscle fibers found that NNT is crucial for mitochondrial H_2_O_2_ clearance and disabling this redox circuit can lead to the development of metabolic disorders [46]. Therefore, the ability of the mitochondria to clear H_2_O_2_ from the cytosolic environment is likely to be highly dependent on the NADPH-forming capacity of NNT. This would mean that cellular redox balance hinges on the capacity of the mitochondria to form a proton gradient (Figure 2).

## 4. Role of the Mitochondria in Degrading Cytosolic H_2_O_2_ Signals

**Figure 3:**
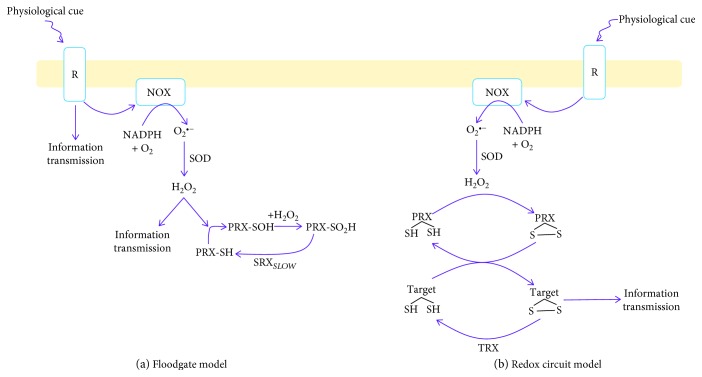
Hydrogen peroxide is a secondary messenger that transmits information in the cytosol using two different mechanisms: the floodgate and redox relay models. The floodgate model involves the activation of cell surface receptors by a physiological stimulus. This induces cell signaling cascades that also activate NADPH oxidase (NOX), activating the production of H_2_O_2_. The hydrogen peroxide yielded from NOX activation, results in the oxidative deactivation of peroxiredoxin-1 (PRX1). Hydrogen peroxide subsequently accumulates, inducing cell signaling pathways or reinforcing others through the deactivation of phosphatases. Reactivation of PRX1 requires sulfiredoxin (SRX). The redox relay model uses a series of thiol disulfide exchange reactions to activate or deactivate a target protein. Hydrogen peroxide generated by a physiological stimulus is first quenched by PRX2 forming a sulfenic acid on the peroxidatic catalytic cysteine. The sulfenic acid is resolved by a second cysteine forming a disulfide bridge. PRX2 is then reduced by STAT3 by a thiol disulfide exchange reaction.

It is now widely recognized that H_2_O_2_ produced by cytosolic enzymes serves as a critical secondary messenger required to regulate a wide range of cellular functions [47]. A variety of hormones including growth factors (platelet-derived growth factor (PDGF), epidermal growth factor (EGF), insulin, and IGF) and cytokines (tumour necrosis factor-*α*; TNF*α*, and angiotensin II) can stimulate O_2_^•−^ production by nonimmune cell NADPH oxidases (NOX) [47]. The O_2_^•−^ formed by NOXs is then rapidly converted to H_2_O_2_ by SOD for the induction of oxidative eustress signaling pathways [48]. Hydrogen peroxide signals emanating from cytosolic enzymes have been proposed to proceed via two mechanisms: (1) the floodgate model and (2) the redox relay model (Figure 3). Empirical evidence has demonstrated that both systems are used by mammalian cells for information transmission. Work on the floodgate model can be traced back to a landmark study by Irani et al. where it was shown that PDGF can stimulate cellular H_2_O_2_ production in vascular smooth muscle cells leading to the induction of chemotaxis and DNA synthesis [49]. Later studies demonstrated that this was associated with increased growth factor receptor phosphorylation, which was related to the H_2_O_2_-mediated deactivation of the protein tyrosine phosphatases [50–52]. The accumulation of H_2_O_2_ for signaling was found to be facilitated by the temporary oxidative deactivation of cytosolic peroxiredoxin (PRX1) [52]. This involves the oxidation of the catalytic Cys_P_ to sulfinic acid (SO_2_H) through a sulfenic acid intermediate (SOH). The SO_2_H can be reduced back to its corresponding thiol by sulfiredoxin (SRX), but the reaction is slow (Figure 3) [53]. This allows H_2_O_2_ to accumulate to a high enough concentration to serve as a secondary messenger. The redox relay mechanism basically involves a series of disulfide exchange reactions between PRX and a target protein (Figure 3). For instance, Sobotta et al. observed that the cytokine-mediated activation of STAT3, and its subsequent deactivation, involves a series of disulfide exchange reactions with oxidized PRX2 and TRX1 [54]. In this case, cytokine signaling induces a burst in H_2_O_2_ generation resulting in its degradation by the peroxidatic cysteine in PRX2 [54]. The resulting disulfide bridge formed between the peroxidatic cysteine and the resolving cysteine in PRX2 is reduced by STAT3, activating the protein [54]. This disulfide bridge is then reduced by TRX1 in the presence of NADPH, deactivating STAT3.

**Figure 4:**
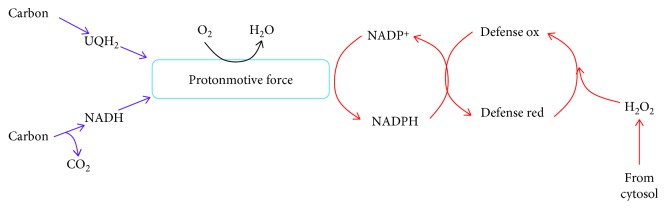
The proton gradient plays a central role in the cellular ROS stabilizing function of the mitochondria. Carbon oxidation forms the electron carriers, NADH and UQH_2_, which are then oxidized by the electron transport chain. Electron flow creates a proton motive force which is tapped by transhydrogenase for the provision of NADPH for antioxidant defenses which degrades cellular H_2_O_2_ signals.

It is not known if mitochondrial antioxidant defenses are required to degrade cytosolic H_2_O_2_ signals formed by NOX (or other cytosolic enzymes) even though interactions between the mitochondria and NOX have been documented. For instance, mitochondria have been found to crosstalk with NOX through a process called “*feed forward cycling*” [55]. This mechanism involves crosstalk between NOX and the mitochondria resulting in the activation of ROS production from both sources. Mitochondria have several properties that allow it to serve as a sink for cellular H_2_O_2_ signals. First, respiring mitochondria can generate NADPH quickly. NNT is a major supplier for NADPH and plays a critical role in quenching cellular H_2_O_2_. When NNT is active, liver mitochondria can replenish matrix NADPH levels within a few minutes [31]. However, deletion of the *Nnt* gene induces a considerable lag in NADPH recovery in actively respiring mitochondria [31]. In addition, Dey et al. showed that NNT is a major NADPH supplier in cultured cells and plays an important role in cellular H_2_O_2_ degradation [28]. As noted in Section 3, several other enzymes can tap into various carbon sources to produce NADPH. However, using the proton gradient for NADPH production is advantageous since it is a readily accessible form of sustainable energy that can be produced by the oxidation of a number of different carbon sources (Figure 2). Therefore, the capacity of the mitochondria to degrade cellular H_2_O_2_ signals may ultimately rely on the PMF and polarity of the mitochondrial inner membrane (Figure 4). A second important consideration is the resistance of PRX3 and 5 towards oxidative deactivation and the abundance of GSH in the matrix. The redox relay and floodgate systems in the cytosol rely on the temporary oxidative deactivation of PRX1 and 2 for signal propagation. The caveat to this mechanism is the hindrance of an important H_2_O_2_ clearing system in the cytosol. Disabling a PRX1 and 2 could be dangerous since it can prolong redox signals that could potentially damage the cell. Mitochondria are not only enriched in antioxidant defenses but also contain PRX isoforms that are highly resistant to oxidative deactivation. It has been shown that PRX1 can rapidly undergo oxidative deactivation by H_2_O_2_ [56]. The matrix-associated isoform PRX3, on the other hand, is highly resistant to oxidative deactivation [56]. This would mean that the mitochondria can maintain their H_2_O_2_-clearing capacity even when cytosolic systems may be deactivated by high H_2_O_2_ levels. Mitochondria are also rich in GSH (~5 mM) and contain high concentrations of the individual enzymes involved in antioxidant defenses (discussed in Section 3). Finally, liver, cardiac, and potentially muscle mitochondria contain catalase, which can also eliminate cytosolic H_2_O_2_ rapidly. Another important factor is that the thiol oxidoreductase, glutaredoxin-2 (GRX2), can substitute for TR2 if it is deactivated by lipid hydroperoxides and reactivate the TRX2 antioxidant system [57]. Finally, the same factors that induce cytosolic H_2_O_2_ signals have also been documented to activate mitochondrial respiration. Growth factor deprivation hinders mitochondrial respiration [58]. In addition, growth factors like IGF have been found to activate mitochondrial respiration through the induction of the PI3K signaling pathway [59]. Mitochondria are also equipped with regulatory mechanisms, including proton leaks, supercomplex assemblies, and protein S-glutathionylation reactions, that rapidly suppress ROS release from sites of production [60]. By suppressing ROS release, regulatory systems can promote the provision of NADPH for antioxidant defenses and the clearance of H_2_O_2_. Therefore, although hypothetical at this point, mitochondria have a number of redox attributes that could potentially play a significant role in modulating cytosolic H_2_O_2_ signals by facilitating the desensitization of these signals.

## 5. Conclusion

Mitochondria are dynamic organelles that fulfill a myriad of cell functions. This includes serving as a platform for the transmission of cell signals. Mitochondria also serve as a critical source and sink for H_2_O_2_, a secondary messenger that mediates cellular redox signals. Hydrogen peroxide is often dubbed a mitokine since its release from the mitochondria modulates cell functions in response to physiological cues. It is also apparent that the mitochondria have the capacity to serve as a cellular H_2_O_2_ stabilizing device. This is related to the high concentration of antioxidant defense enzymes in the matrix and its capacity to rapidly regenerate NADPH. Here, I have outlined how the ROS quenching capacity of the mitochondria can be utilized to degrade H_2_O_2_ signals emanating from cytosolic enzymes like NOX. Mitochondria are well equipped to serve as a cellular H_2_O_2_ signal dampener. The ability of the mitochondria to quench cellular H_2_O_2_ signals depends on the establishment of a proton motive force to support NADPH production for antioxidant defenses (Figure 4). It is obvious that more research is required to understand how the mitochondria clear H_2_O_2_ from the surrounding environment, the role of NNT in this process, and the function of this ROS dampening feature in controlling cellular redox signals. Based on its bioenergetic properties and high amount of antioxidant enzymes, mitochondria are an excellent candidate for the modulation of cellular H_2_O_2_ signals.

## Figures and Tables

**Table 1:** Mitochondria can contain up to twelve sources of O_2_^•−^/H_2_O_2_. The twelve different enzymes are associated with nutrient metabolism and can be subcategorized in two groups: the NADH/NAD^+^ isopotential group and UQH_2_/UQ isopotential group. The different sites make variable contributions to overall ROS release in different tissues.

Isopotential group	Enzyme	Site of production	High capacity site?
NADH/NAD^+^	*α*-Ketoglutarate dehydrogenase	FAD (K_F_)	Yes: liver, muscle [8, 10] No: cardiac [11]
	Pyruvate dehydrogenase	FAD (P_F_)	Yes: liver, muscle [8] No: cardiac [11]
	Branched-chain keto acid dehydrogenase	FAD (B_F_)	Moderate: muscle [10] Unknown: liver, cardiac.
	2-Oxoadipate dehydrogenase	FAD (O_F_)	Moderate: muscle [10] Unknown: liver, heart
	Complex I	FMN (I_F_)	Yes: cardiac [11] No: muscle, liver [8]

UQH_2_/UQ	Complex I	UQ binding site (I_Q_)	Yes: muscle [10] Unknown: liver, cardiac
	Complex II	FAD (II_F_)	Yes: muscle, liver (129 mice only), cardiac [61]. No: liver (C57Bl6N) [11].
	Complex III	UQ outer leaflet binding site (III_Qo_)	Yes: muscle, liver, cardiac [8]
	Electron transfer flavoprotein: ubiquinone oxidoreductase	FAD (E_F_)	No: muscle [10] Unknown: liver, cardiac
	*sn-*Glycerol-3-phosphate dehydrogenase	FAD (G_F_)	Yes: muscle, liver, cardiac [10]
	Proline dehydrogenase	FAD (P_F_)	No: muscle [10] Unknown: liver, cardiac
	Dihydroorotate dehydrogenase	FAD (D_F_)	No: muscle [10] Unknown: liver, cardiac
